# South London Hospital for Women

**Published:** 1916-02-19

**Authors:** 


### THE EDITOR'S LETTER-BOX.

### Some Interesting Problems.

**Our Correspondents are reminded that brevity of style and conciseness of statements greatly facilitate early insertion. We cannot in any way be responsible for the opinions expressed by correspondents, who must give their name and address as a guarantee of 'good faith, but not necessarily for publication.**

### South London Hospital for Women.

*To the Editor of* The Hospital.

Sir,—With reference to the advertisement appearing in The Hospital, February 12, for matron, South London Hospital for Women, Clapham Common, would you kindly send me some particulars?

The advertisement suggests a new hospital. Who are the governing body? Could you give me any information regarding the hospital that you may possess? Is it likely to be supported by King Edward's Hospital Fund?—Yours truly, Matron.

February 13, 1916.

[If our correspondent will refer to Burdett's "Hospitals and Charities, 1915," she will find particulars of this hospital. Hospitals within the County of London area which fulfil the conditions laid down by the King's Fund are periodically invited to lay their claims before it.—Ed. The Hospital.]

